# The Treatment of Fractured Patella

**Published:** 1892-10-29

**Authors:** 


### The Practitioners' Mirror and Retrospect.

### SURGERY.

### THE TREATMENT OF FRACTURED PATELLA.

It is only during the last half century that transverse fractures of the patella have been treated by fastening the fragments together; and since antiseptic surgery became established the accident can no longer be considered to have an unfavourable prognosis as regards the utility of the limb. Before this the results were very unsatisfactory, and almost every eminent surgeon brought his ingenuity to bear on the subject and used some special method of treatment. "Clustered round this simple lesion," writes Mr. Arthur E. Barker, "is a veritable galaxy of surgical luminaries, all concentrating their brilliant light upon it."

The following is a short outline of the more important recent plans of treatment:—

Mr. T. A. Cabot narrates (in the *Boston Medical and Surgical Journal* for November 19th, 1891) three cases of compound fracture of the patella, in which he wired together the fragments and obtained good results. He did not advocate this in simple fractures, on the ground that a useful limb results even if there is only fibrous union and the two fragments are separated by a wide interval. This opinion is not generally held; in fact, the very unsatisfactory condition of the limb after ordinary treatment has long been an established fact.

Mr. Arthur E. Barker gave a clinical lecture at University College Hospital early in the present year (reported in the *British Medical Journal* for February 27th) on four cases of recent transverse fracture of this bone, which he had treated as follows:—A cut was made below the patella, in the middle line, and at the spot where the fibres of the ligamentum patella are connected with that bone. Through this a stout pedicle needle, threaded with strong silk or wire, was introduced upwards through the joint. A second incision was then made immediately above the patella, and the point of the needle thrust through this, the wire caught and the needle withdrawn. The wire was therefore behind the bone. Again the needle was introduced through the first (lower opening) and out through the second (higher) one, but this time superficial to the bone. The wire was threaded into the eye, and when the needle was again withdrawn the upper and lower fragments were thus encircled with a wire or silk loop, which was knotted just below the bone. The fragments were previously rubbed together, and blood clots squeezed out through the openings. An antiseptic dressing was applied, and an ice-bag over the knee. The splint was removed in ten days. At the end of five weeks the patients could walk freely. In some of these cases the most violent and trying movements of the leg could be indulged in with impunity after the operation.

M. Berger describes (in *La Semaine Médicale*, July 19th, 1892) an operation on one of these fractures in which the plaster stocking had been previously tried without success. He cut down on the patella, scraped the surfaces, and brought them into apposition. He then passed a silver wire transversely through the insertion of the quadriceps extensor, round the bone, and through the ligamentum patella close to the lower fragment. When the wires were tightly twisted there was close juxtaposition of the fragments, and the case is stated to have been most successful.

Comparing this with the method of Mr. Barker, we notice that there is more exposure of the bone, but nothing is actually introduced into the joint cavity.

Mr. Herbert Butcher has criticised Mr. Barker's procedure, and recommends an operation on the same lines as M. Berger, except that it is entirely subcutaneous. No wire or needle is allowed to enter the joint cavity, and the patella is encircled with its wire loop from two small lateral skin openings. This method appears to have much to recommend it; it is simple, fairly easy, and entirely subcutaneous. The objections raised, however, to Mr. Barker's plan do not seem to be valid; as a matter of fact there has been no discomfort from the position of the knot, and no evil result from the presence of the ligature behind the patella.

Mr. D. W. Aitken (*Medical Journal*, July 23rd, 1892) points out that in the above plans there may possibly be a want of firm apposition. He therefore recommends an operation as follows: The knee is slightly bent, the fractured bone fixed, and then a drill, threaded near the end with wire, is thrust through the patella from below upwards, not encroaching on the joint, and piercing the skin at the upper border. The wire is pulled out, and the drill withdrawn. It is then again inserted at the first opening (below the patella) and brought out at the second opening (above), but this time superficial to the bone. The wire is again threaded through the eye of the drill, and the latter withdrawn. The patella is, therefore, pierced with the wire, which does not enter the joint.

Mr. Aitken claims for this plan that it is easy of performance, that it is entirely subcutaneous, and that the fragments are firmly united.

Other and more complicated operations have been suggested and performed, but the above have so much to commend them, that in future it will probably be a question of the best means of fastening the fragments together with as little exposure of the bone or other structures as possible; the older methods of Malgaigne's hooks, arrangements with strapping or plaster of Paris, special splints, &c., have had their day.

There is one point, however, that must be borne in mind: no surgeon should undertake any operation of this kind, even a purely subcutaneous one, unless he is competent to carry out the strictest antiseptic precautions. The slightest risks must be avoided. If there is any doubt on this point, one of the older plans must be adopted. Fortunately, it is not difficult to ensure asepsis. The joint should be previously well soaped and washed, rubbed with permanganate of potash solution (and then with a solution of oxalic acid), or with some other decided antiseptic lotion. The drill or needle, &c., should be made absolutely pure, and the surgeon's hands attended to. Any carelessness on these points might compromise the operation or even endanger the patient's life.

If the surgeon is certain to ensure perfect asepsis, probably Mr. Barker's method is the most promising, for the two incisions enable the blood clots and synovial membrane, &c., to be dislodged from between the fragments more effectively than the purely subcutaneous plans.

